# Significant reduction in antibiotic prescription rates in Japan following implementation of the national action plan on antimicrobial resistance (2016–20): a 9-year interrupted time-series analysis

**DOI:** 10.1093/jacamr/dlaf062

**Published:** 2025-04-30

**Authors:** Hideki Hashimoto, Naoki Kanda, Hiromasa Yoshimoto, Kazuo Goda, Naohiro Mitsutake, Shuji Hatakeyama

**Affiliations:** Division of General Internal Medicine, Jichi Medical University Hospital, 3311-1 Yakushiji, Shimotsuke-shi, Tochigi 329-0498, Japan; Hitachi Social Cooperation Education Research Centre, University of Tsukuba Hospital, 2-1-1 Jonancho, Hitachi-shi, Ibaraki 317-0077, Japan; Division of General Internal Medicine, Jichi Medical University Hospital, 3311-1 Yakushiji, Shimotsuke-shi, Tochigi 329-0498, Japan; Department of Research, Institute for Health Economics and Policy, 1-5-11, Nishi-Shimbashi, Minato-ku, Tokyo 105-0003, Japan; Institute of Industrial Science, The University of Tokyo, 4-6-1 Komaba, Meguro-ku, Tokyo 153-8505, Japan; Department of Research, Institute for Health Economics and Policy, 1-5-11, Nishi-Shimbashi, Minato-ku, Tokyo 105-0003, Japan; Division of General Internal Medicine, Jichi Medical University Hospital, 3311-1 Yakushiji, Shimotsuke-shi, Tochigi 329-0498, Japan; Division of Infectious Diseases, Department of Infection and Immunity, Jichi Medical University, 3311-1 Yakushiji, Shimotsuke-shi, Tochigi 329-0498, Japan

## Abstract

**Background:**

Research on the effectiveness of Japan’s national action plan on antimicrobial resistance, including among individuals with HIV, remains scarce.

**Objectives:**

To evaluate the impact of policies on antibiotic prescription practices.

**Methods:**

Outpatient oral antibiotic prescription data from 2012 to 2020 were extracted from a national claims database comprising >98% of the Japanese population. Prescription rates were stratified according to antibiotic class, diagnosis and HIV status. An interrupted time-series analysis was performed to assess the impact of the national action plan.

**Results:**

An average of 129,989,400 prescriptions were issued annually (1024 per 1000 population-years). Between 2012 and 2020, the oral antibiotic prescription rate decreased by 54%. The prescription rate showed a significant downward trend post-intervention (additional annual reduction in incidence rate ratio, 0.889; 95% confidence interval, 0.889–0.990). However, broad-spectrum antibiotics (third-generation cephalosporins, macrolides and fluoroquinolones) remained prevalent, comprising 84.7% and 71.4% of prescriptions in 2012 and 2020, respectively. Antibiotic prescriptions during outpatient visits for pharyngitis, sinusitis, bronchitis and viral upper respiratory infections decreased significantly (rate ratios = 0.66, 0.76, 0.51 and 0.49, respectively). The antibiotic prescription rate was ∼2.5-fold higher in individuals with HIV than in those without.

**Conclusions:**

Antibiotic prescription rates significantly decreased following the implementation of the national action plan. However, a sharp decline in 2020, likely due to the coronavirus disease pandemic, requires continued rebound monitoring. Reducing broad-spectrum oral antibiotic overuse remains a critical focus.

## Introduction

Antimicrobial resistance (AMR) threatens global health, leading to increased morbidity and mortality. Consequently, numerous countries have adopted national action plans to mitigate AMR through prudent antibiotic use.^1^ The WHO AWaRe classification system for promoting appropriate antibiotic use categorizes antibiotics into three groups: access, watch and reserve.^2^ Among these, the ‘access’ antibiotics comprise narrow-spectrum agents with a low risk of inducing resistance (e.g. amoxicillin). Various countries are striving to increase the use of this class of antibiotics.

An analysis of oral antibiotic prescriptions in Japanese outpatient settings from 2012 to 2015 showed the extensive use of broad-spectrum antibiotics, specifically third-generation cephalosporins, macrolides and quinolones. Furthermore, 56% of all antibiotic prescriptions were for conditions with limited or no indication for antimicrobial therapy, including bronchitis and viral upper respiratory infections (URIs).^3^ The Japanese government launched the national action plan on AMR in 2016 to combat inappropriate antibiotic use. The plan included key elements, such as raising public awareness of AMR, monitoring antimicrobial use and promoting the appropriate use of antibiotics. As outcome measures, the plan aimed to reduce overall antibiotic use to two-thirds of the 2013 levels by 2020, with targeted reductions in oral cephalosporins, macrolides and fluoroquinolones by 50%.^4^ The Ministry of health, labour and welfare (MHLW) introduced an antimicrobial stewardship manual and financial incentives to enhance antimicrobial stewardship initiatives to achieve these goals.^5,6^

Several studies have evaluated the effectiveness of Japan’s national action plan; however, its overall impact on AMR remains ambiguous due to varying outcomes and the omission of older populations in many analyses.^7–9^ This may result in a biased evaluation of the plan’s impact across different age groups. Moreover, research is lacking on specific populations at higher risk of antibiotic over-prescription, including individuals with HIV infection.^10^

Therefore, the present study aimed to evaluate the effectiveness of Japan’s national action plan by leveraging comprehensive database resources and analysing antibiotic prescription trends, including those of people with HIV (PWH) over time. Such investigations are vital for understanding the tangible impacts of policy measures on antimicrobial prescription practices and developing strategies to mitigate AMR.

## Materials and methods

### Ethics

This study complied with the principles of the Declaration of Helsinki and was approved by the Ethics Committee of Jichi Medical University Hospital, Tochigi, Japan (approval number 22-082). Owing to the retrospective nature and anonymization of the data, the Ethics Committee waived the requirement for informed consent.

### Data sources and the health insurance system in Japan

Our sequential cross-sectional analysis used data from the National Database of Health Insurance Claims and Specific Health Check-ups of Japan (NDB) between April 2012 and March 2021. The NDB is a nationwide administrative claims database established by the MHLW of Japan. This database includes claims data of all eligible Japanese citizens under insurance programmes and the public assistance system.^11^ In Japan, universal coverage is provided through three insurance programmes: National Health Insurance System (for self-employed individuals, unemployed individuals aged <75 years and their dependents), Employee Health Insurance System (for employees aged <75 years and their dependents) and Elderly Health Insurance System (for individuals aged ≥75 years). Furthermore, within the public assistance system, the government fully subsidizes the medical expenses of individuals receiving welfare benefits who are experiencing financial hardships.

The NDB encompasses medical and pharmacy claims that can be linked to unique anonymized identifiers. It includes data on sex, age, diagnostic codes with dates of diagnoses, medical procedure codes with dates of procedures, drug codes with dates of prescriptions and the prefecture in which the medical facility is located. Laboratory test results and the characteristics of prescribing physicians are not included in the claims database. The diagnostic codes are classified according to the International Classification of Diseases and Related Health Problems 10th Revision (ICD-10). As of 2012, 99% of claims from hospitals and pharmacies in Japan were submitted electronically. For clinics, electronic submission rates were 93% in 2012, increasing to 98% by 2015.^12^

### Data preparation

We linked medical and pharmacy claims using a unique identifier derived from a combination of birth date, sex and name. We collected information on all oral antibiotics prescribed in medical outpatient settings using aggregated claims data. Antibiotics were classified according to the WHO Anatomical Therapeutic Chemical system^13^ or Japan’s AMR Clinical Reference Centre classification system.^14^

We also identified all outpatients diagnosed with infectious diseases. These diagnoses were categorized into 20 groups and three categories based on the indication for antibiotic use (Table S1, available as Supplementary data at *JAC-AMR* Online).^3^ To evaluate the appropriateness of the antibiotic prescriptions, the prescriptions were linked to diagnoses when both were documented on the same date. In Japan’s health insurance system, antibiotic costs cannot be reimbursed without a corresponding infectious disease diagnosis, significantly reducing the likelihood of antibiotic prescription without a legitimate diagnosis. For instances of multiple infectious disease diagnoses on a single day, the diagnoses were prioritized in the order of Group 1 (where antibiotics are usually indicated), Group 2 (where antibiotics are potentially indicated) and Group 3 (where antibiotics are rarely indicated). Because diagnostic codes were recorded only at the initial visit for each illness episode, information on antibiotic prescriptions at subsequent follow-up visits was not linked to diagnoses.

### Data analyses

We first analysed the national trends in antibiotic prescriptions from April 2012 to March 2021, especially the changes in trends after the implementation of the national action plan in 2016. We defined antibiotic prescription rates as the number of prescriptions per 1000 individuals, calculated monthly or annually and stratified by prefecture, diagnosis and antibiotic category. Each antibiotic was counted independently when multiple antibiotics were prescribed on the same day.

We also calculated the proportions of outpatient visits for infectious diseases that resulted in antibiotic prescriptions, categorized by diagnosis, to evaluate changes in physicians’ prescribing behaviours. The numerator was the number of outpatient visits in which an antibiotic was prescribed for each infection. At the same time, the denominator was the total number of outpatient visits with a diagnosis of each infection. Multiple antibiotics prescribed on the same day were considered a single prescription in the sub-analysis. For pharyngitis and sinusitis, we calculated the proportions of antibiotics recommended in the guidelines as described previously^3^ and described the annual trends.

We also analysed trends in antibiotic prescription rates between individuals with and without HIV infection and stratified by diagnosis. Each year, individuals were identified as PWH if they met two criteria: having a prior or current HIV diagnosis and either receiving antiretroviral therapy or undergoing tests for HIV-1 RNA or CD4+ T lymphocyte counts within that year. HIV diagnoses were determined using the ICD-10 codes B20–24 and Z21.

Data from groups with <10 individuals were masked according to NDB guidelines to protect personal information. Prescription rates were standardized to the 2015 national population^15^ and tabulated according to sex and age. The age and sex distributions were standardized to the demographics of Japan in 2015 to calculate annual prescriptions per 1000 patients. The rates among PWH were standardized to the 2015 PWH population for comparison with individuals without HIV (PWoH), who were also standardized to the 2015 PWH population.

### Statistical analysis

The 9-year change in antibiotic prescription rates was estimated using the rate ratio between fiscal years 2012 and 2020, along with the corresponding confidence interval (CI), through a generalized linear regression with a Poisson distribution. A Poisson regression model was used to outline the overall trends in antibiotic use throughout the study period. This model used the number of months as the primary factor and was adjusted for seasonal variation.

We conducted an interrupted time-series analysis to assess the effectiveness of the National Action Plan in reducing antibiotic use after policy implementation. An interrupted time-series analysis measures the impact of health policies or interventions introduced at a specific time point.^16^ We analysed the changes in antibiotic prescription rates in outpatient settings before (April 2012 to March 2016) and after (April 2016 to March 2021) implementation of the national action plan. We also applied the same analyses to evaluate its effect on the proportion of antibiotic prescriptions for specific respiratory tract infections, including pharyngitis, sinusitis, bronchitis and viral URIs. Additionally, to mitigate the impact of coronavirus disease (COVID-19), we conducted a sub-analysis that omitted fiscal year 2020 from the post-implementation period. All statistical analyses were performed using R software (version 4.1.2; R Foundation for Statistical Computing, Vienna, Austria) with a two-tailed hypothesis test at a 5% significance level.

## Results

### Trends in overall oral antibiotic prescription rates

**Figure 1.**
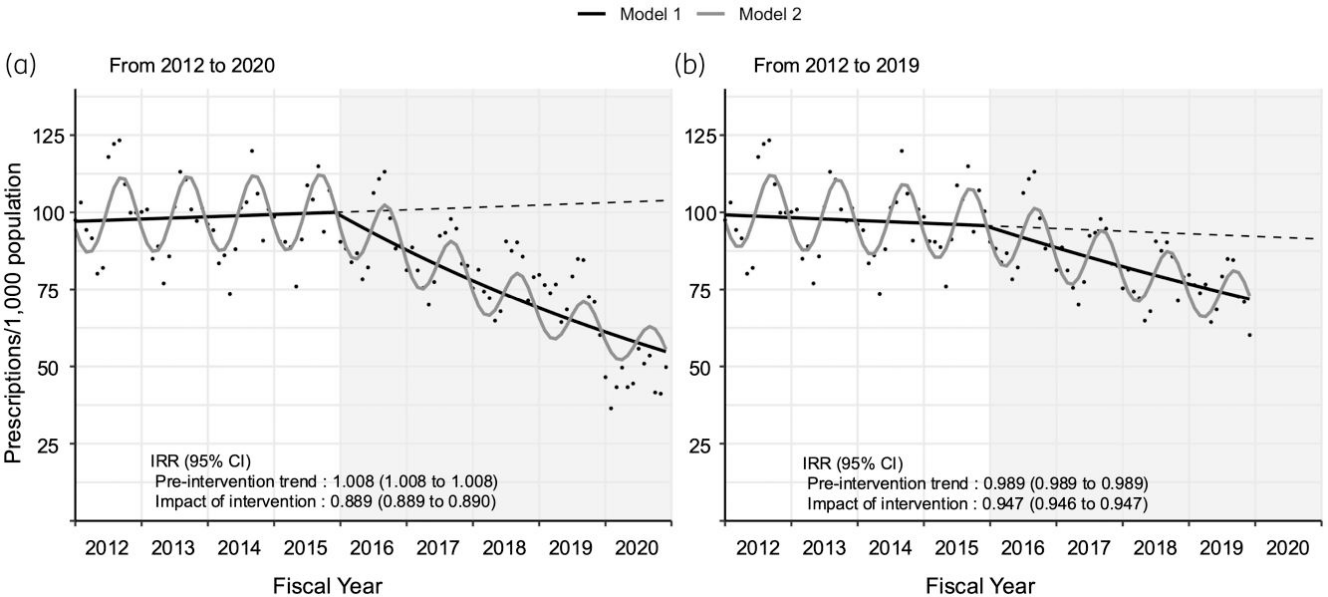
Trends and interrupted time-series analyses of the overall oral antibiotic prescription rates per 1000 individuals in Japan. (a) Main analysis between fiscal years 2012 and 2020. (b) Sub-analysis between fiscal years 2012 and 2019 with the exclusion of the coronavirus disease 2019 (COVID-19) pandemic period. Vertical line: timing of National Action Plan implementation in April 2016. Grey background colour: post-intervention period. Dots: monthly antibiotic prescription rates. Solid lines: estimated model. Model 1 (black line): unadjusted linear regression. Model 2 (grey line): linear regression adjusted for seasonality. Dashed lines: counterfactual scenario without the intervention. The IRR and its 95% CI of the pre-intervention trend were estimated using Poisson regression to reflect changes in the incidence rate between 2012 and 2016. The impact of intervention represents the additional annual reduction in the IRR during the post-intervention period (2016–2020).

During the 9-year study period, an average of 129,989,400 prescriptions were issued annually (1024 prescriptions per 1000 population-years). Of these, 66% (655 per 1000 population-years) were linked to an infection diagnosis. Between 2012 and 2020, the annual antibiotic prescription rate decreased by 54%, from 1221 to 556. Before the intervention, defined as the implementation of the national action plan in April 2016, prescription rates increased annually by 0.8% (incidence rate ratio [IRR], 1.008; 95% CI, 1.008–1.009). After the intervention (2016–2020), this trend reversed to an exponential annual decline of 11.1% (IRR, 0.889; 95% CI, 0.889–0.890) (Table 1). The interrupted time-series analysis showed a significant decline in the overall trend of antibiotic prescription rates before and after the intervention (*P *< 0.01, Figure 1a). A sub-analysis that excluded 2020, the first year of the COVID-19 pandemic, also demonstrated a similar trend, albeit with a lower reduction in prescription rates (*P *< 0.01, Figure 1b).

**Table 1.** Number of oral antibiotic prescriptions per 1000 population by antibiotic class

	FY 2012	FY 2013	FY 2014	FY 2015	FY 2016	FY 2017	FY 2018	FY 2019	FY 2020	Pre-intervention trend, IRR (95% CI)^a^	Impact of intervention, IRR (95% CI)^b^
Overall	1221.1	1162.8	1144.1	1164.6	1116.0	1018.6	940.5	891.9	556.3	1.008 (1.008 to 1.009)	0.889 (0.889 to 0.890)
Antibiotic class (%)^c^											
Third cephem	420.7 (34.5)	408.9 (35.2)	404.4 (35.3)	402.8 (34.6)	375.8 (33.7)	342.8 (33.7)	301.8 (32.1)	271.6 (30.5)	166.3 (29.9)	1.010 (1.010 to 1.010)	0.862 (0.862 to 0.862)
Macrolides	382.5 (31.3)	353.2 (30.4)	340.7 (29.8)	349.2 (30.0)	339.0 (30.4)	296.2 (29.1)	268.2 (28.5)	249.4 (28.0)	132.3 (23.8)	1.003 (1.003 to 1.003)	0.870 (0.870 to 0.870)
Quinolones	230.8 (18.9)	219.0 (18.8)	214.9 (18.8)	221.9 (19.1)	212.8 (19.1)	193.5 (19.0)	178.7 (19.0)	167.5 (18.8)	98.6 (17.7)	1.014 (1.014 to 1.015)	0.878 (0.878 to 0.878)
Penicillins	80.5 (6.6)	83.4 (7.2)	86.5 (7.6)	92.3 (7.9)	89.9 (8.1)	92.6 (9.1)	97.4 (10.4)	105.5 (11.8)	66.9 (12.0)	1.063 (1.063 to 1.064)	0.921 (0.921 to 0.922)
Tetracyclines	35.0 (2.9)	30.3 (2.6)	28.9 (2.5)	29.8 (2.6)	31.0 (2.8)	28.8 (2.8)	29.9 (3.2)	31.3 (3.5)	32.5 (5.8)	0.950 (0.949 to 0.950)	1.076 (1.075 to 1.076)
SUL-TMP	10.8 (0.9)	12.1 (1.0)	13.2 (1.2)	14.5 (1.2)	15.8 (1.4)	17.2 (1.7)	18.5 (2.0)	20 .0 (2.2)	20.8 (3.7)	1.106 (1.106 to 1.107)	0.972 (0.971 to 0.973)
Second cephem	17.9 (1.5)	17.1 (1.5)	16.5 (1.4)	16.1 (1.4)	15.6 (1.4)	14.8 (1.4)	15.1 (1.6)	16.6 (1.9)	15.6 (2.8)	0.955 (0.955 to 0.955)	1.047 (1.046 to 1.048)
First cephem	5.6 (0.5)	5.5 (0.5)	5.5 (0.5)	5.6 (0.5)	5.7 (0.5)	6.2 (0.6)	6.9 (0.7)	8.5 (1.0)	8.5 (1.5)	0.982 (0.982 to 0.983)	1.123 (1.121 to 1.124)
Penems	17.4 (1.4)	16.7 (1.4)	16.9 (1.5)	16.5 (1.4)	15.1 (1.4)	13.3 (1.3)	11.8 (1.3)	10.4 (1.2)	7.2 (1.3)	1.002 (1.001 to 1.002)	0.866 (0.865 to 0.866)
Lincosamides	1.2 (0.1)	1.1 (0.1)	1.1 (0.1)	1.1 (0.1)	1.0 (0.1)	1.0 (0.1)	1.0 (0.1)	1.1 (0.1)	0.9 (0.2)	0.968 (0.966 to 0.970)	1.016 (1.013 to 1.019)
Other	18.7 (1.5)	15.6 (1.3)	15.4 (1.3)	14.9 (1.3)	14.5 (1.3)	12.3 (1.2)	11.3 (1.2)	10.0 (1.1)	6.8 (1.2)	0.956 (0.956 to 0.957)	0.921 (0.921 to 0.922)

FY, fiscal year; IRR, incidence rate ratio; CI, confidence interval; Third cephem, third-generation cephalosporins second cephem, second-generation cephalosporins; First cephem, first-generation cephalosporins; SUL-TMP, sulfonamides and trimethoprim.

^a^Changes between 2012 and 2016 were estimated using ratios and 95% confidence intervals [CIs] intervals with Poisson regression.

^b^Additional annual reduction in the IRR during the post-intervention period (2016–20).

^c^Antibiotics were categorized according to Anatomical Therapeutic Chemical (ATC) codes: Penicillins, J01C; first-generation cephalosporins, J01DB; second-generation cephalosporins, J01DC; third-generation cephalosporins, J01DD; penems, J01DH and J01DI; macrolides, J01FA; lincosamides, J01FF; quinolones, J01M; sulfonamides and trimethoprim, J01E; tetracyclines, J01A; other antibiotics, J01B, J01G and J01X.

Among prescribed oral antibiotics, third-generation cephalosporins comprised the highest proportion (35% in 2012 and 30% in 2020), followed by macrolides (31% in 2012 and 24% in 2020) and fluoroquinolones (19% in 2012 and 18% in 2020) (Table 1). Broad-spectrum antibiotics (third-generation cephalosporins, macrolides and fluoroquinolones) accounted for 85% and 71% of the total use in 2012 and 2020, respectively. The prescription rate of penicillins increased from 7% in 2012 to 12% in 2020, a modest increase of 5%.

Oral antibiotic prescription rates in Japan exhibited a trend of being higher in the western regions and lower in the eastern regions (Table S2). In 2013, the prefectures with the highest antibiotic prescription rates were Kumamoto (1393 per 1000 individuals), Oita (1390) and Gifu (1377); whereas, the lowest rates were observed in Hokkaido (945), Yamagata (954) and Iwate (969). Despite the overall decrease in antibiotic prescriptions, regional disparities remained evident in 2020, with Miyazaki (795), Oita (742) and Saga (717) have the highest rates and Hokkaido (451), Chiba (455) and Saitama (468) having the lowest rates.

### Trends in antibiotic prescriptions for specific infectious diagnoses

The number of oral antibiotic prescriptions per 1000 population for Group 1 infections, including urinary tract infections, pneumonia, abdominal infections and sexually transmitted infections, remained relatively stable over the study period, except for 2020. Among Group 2 infections, the oral antibiotic prescription rates per 1000 individuals for pharyngitis and gastrointestinal infections showed a decreasing trend after 2016. However, the incidence of sinusitis, skin and soft tissue infections, suppurative otitis media and acne remained stable or increased slightly by 2019. For Group 3 infections, including bronchitis and viral URIs, oral antibiotic prescription rates consistently decreased after 2016. Notably, the antibiotic prescription rate for respiratory infections, including pneumonia, pharyngitis, sinusitis, suppurative or non-suppurative otitis media, bronchitis, viral URIs, influenza, gastrointestinal infections and trauma and burns markedly decreased in 2020 owing to the impact of the COVID-19 pandemic (Table 2).

**Table 2.** Number of antibiotic prescriptions per 1000 population for specific infections

	FY 2012	FY 2013	FY 2014	FY 2015	FY 2016	FY 2017	FY 2018	FY 2019	FY 2020
Overall	740.7	725.3	732.6	758.3	738.1	678.8	621.1	585.5	316.1
Group 1	69.6	68.4	70.8	78.8	79.0	74.5	73.0	73.2	53.4
Urinary tract infections	35.0	35.5	35.8	36.4	35.9	35.2	34.7	34.7	32.8
Pneumonia	13.7	11.9	11.9	14.3	16.2	12.5	11.9	11.8	4.9
Abdominal infections	1.8	1.8	1.9	2.0	2.0	2.0	2.0	2.0	2.0
Sexually transmitted infections	1.8	1.9	1.9	2.0	2.0	2.1	2.2	2.3	2.2
Miscellaneous bacterial infection	17.3	17.3	19.3	24.1	22.9	22.7	22.2	22.4	11.5
Group 2	286.4	292.6	304.0	318.2	315.1	302.7	288.8	279.1	160.6
Pharyngitis	138.9	139.4	142.3	147.5	144.6	134.2	119.4	112.8	50.2
Sinusitis	81.8	84.9	90.4	95.8	95.0	94.2	92.9	89.2	43.8
GI infections	2.9	2.5	2.6	2.6	2.5	2.3	2.2	2.0	1.4
Skin infections	33.5	35.6	36.9	39.1	39.8	40.0	41.3	42.6	41.2
Suppurative otitis media	20.8	21.1	22.3	23.5	23.5	22.0	21.8	20.5	9.9
Acnes	8.5	9.1	9.5	9.7	9.7	10.0	11.2	12.0	14.1
Group 3	392.5	372.4	367.8	373	356.2	313.3	271.3	244.3	103.3
Bronchitis	188.6	176.9	173.5	178	168.7	144.1	124.1	111.5	40.7
Viral URI	155.6	147.7	144.5	144.8	137.7	121.0	103.2	93.3	40.7
Trauma and burn	20.8	21.1	22.3	23.5	23.5	22.0	21.8	20.5	9.9
Eye infection	13.1	12.3	12.7	12.4	11.9	11.6	11.4	10.3	8.3
Influenza	7.3	7.4	7.7	7.0	6.9	7.6	4.4	2.8	0.1
Fever	4.5	4.5	4.6	4.8	5.1	4.8	4.3	4.1	2.6
Non-suppurative otitis media	2.4	2.3	2.3	2.3	2.2	2.0	1.9	1.7	0.9
Non-bacterial GI infections	0.2	0.2	0.2	0.2	0.2	0.2	0.2	0.1	0.1
Viral pneumonia	0	0	0	0	0	0	0	0	0

FY, fiscal year; GI, gastrointestinal infection; URI, upper respiratory infection.

Groups 1, 2 and 3: infections for which antibiotics are usually, potentially and rarely indicated, respectively.

**Figure 2.**
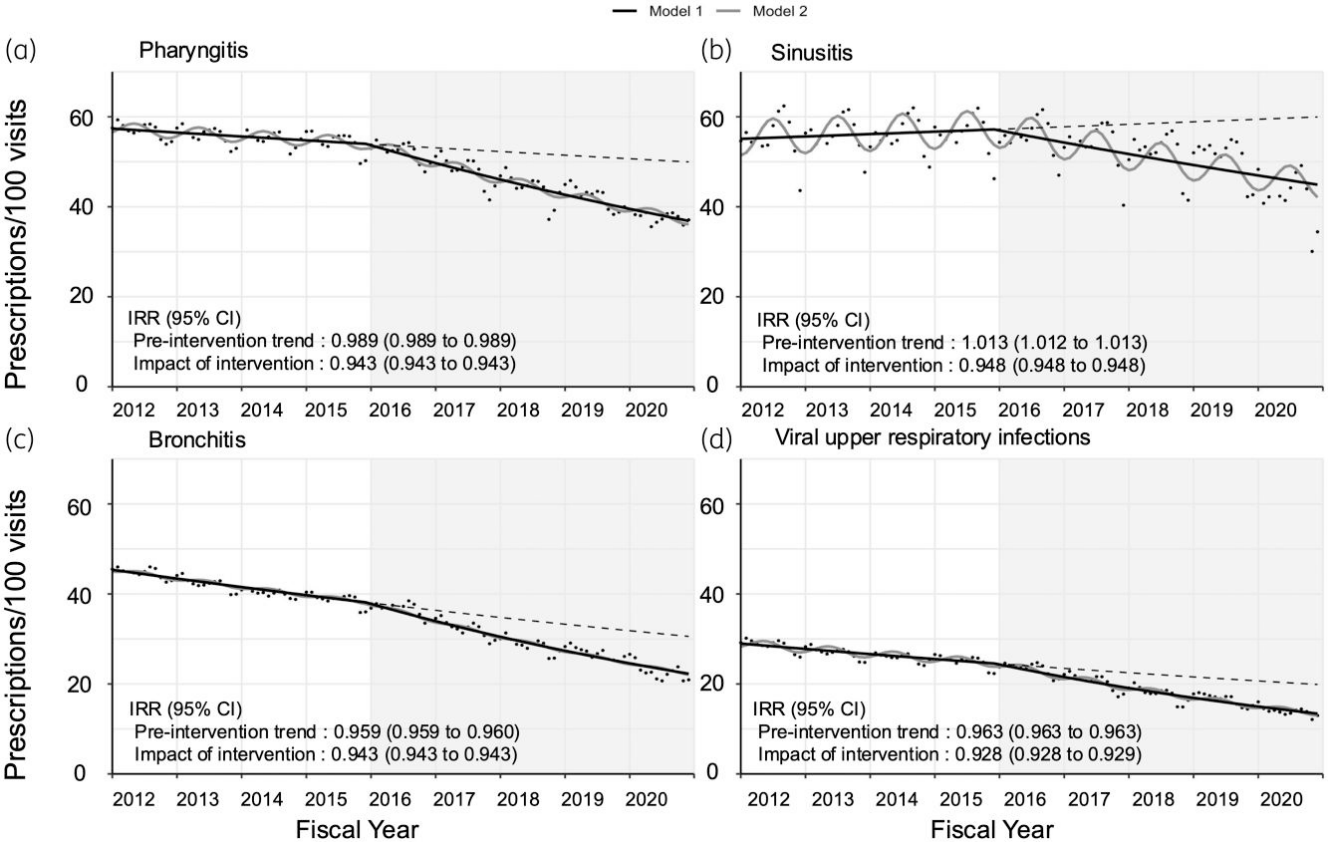
Trends and interrupted time-series analyses of the proportions of antibiotic prescriptions for specific infections in Japan between fiscal years 2012 and 2020. Antibiotic prescriptions for (a) pharyngitis, (b) sinusitis, (c) bronchitis and (d) viral URIs. Vertical line: timing of National Action Plan implementation in April 2016. Grey background colour: post-intervention period. Dot: monthly numbers of antibiotic prescriptions per 100 visits for each infection (proportion of prescribing). Solid lines: estimated model. Dashed lines: counterfactual scenario without the intervention. The IRR and its 95% CI of the pre-intervention trend were estimated using Poisson regression to reflect changes in the incidence rate between 2012 and 2016. The impact of intervention represents the additional annual reduction in the IRR during the post-intervention period (2016–2020).

The proportion of antibiotic prescriptions for specific infections during outpatient visits significantly decreased after 2016 (Figure 2 and Table S3). For pharyngitis, this proportion decreased from 56.9% in 2012 to 37.7% in 2020 (rate ratio, 0.66). Similarly, that for sinusitis decreased from 55.2% in 2012 to 41.9% in 2020 (rate ratio: 0.76) that for bronchitis from 44.7% in 2012 to 22.6% in 2020 (rate ratio: 0.51), and that for viral URIs decreased from 28.5% in 2012 to 13.9% in 2020 (rate ratio: 0.49).

In contrast, the proportion of gastrointestinal infections did not decrease during the study period (36.3% and 41.2% in 2012 and 2020, respectively; rate ratio, 1.13). The results of the interrupted time-series analysis showed a significant decrease in the proportion of antibiotic prescriptions for pharyngitis, sinusitis, bronchitis and URIs after 2016 *(P *< 0.01) (Figure 2). A sub-analysis excluding data from 2020 showed trends similar to the main analyses for these four infections (*P *< 0.01) (Figure S1), indicating the minimal impact of the COVID-19 pandemic on the proportion of antibiotic prescriptions for the treatment of respiratory infections.

### Proportions of first-line antibiotic use for pharyngitis and sinusitis

The proportions of first-line antibiotics (penicillin) prescribed for pharyngitis and sinusitis increased from 7.7% in 2012 to 16.2% in 2020 (rate ratio: 2.10) and from 10.5% in 2012 to 16.5% in 2020 (rate ratio: 1.57), respectively. However, broad-spectrum antibiotics constituted the majority (>80%) of prescriptions throughout the study period. For pharyngitis, the proportion of third-generation cephalosporins decreased modestly from 42.9% in 2012 to 38.0% in 2020 (rate ratio, 0.89) and that of macrolides from 27.6% to 23.0% (rate ratio, 0.83), whereas that of fluoroquinolones did not decrease, remaining at 18.1% in 2012 to 19.1% in 2020 (rate ratio, 1.06). A similar trend was observed for sinusitis (Table 3).

**Table 3.** Proportions of first-line and non-first-line antibiotics prescribed for pharyngitis and sinusitis

	FY 2012	FY 2013	FY 2014	FY 2015	FY 2016	FY 2017	FY 2018	FY 2019	FY 2020
Pharyngitis (%)									
First-line agent (penicillins)	7.7	8.0	8.5	8.5	8.7	10.0	11.7	13.5	16.2
Non-first-line agent									
Third cephem	42.9	43.3	43.5	42.8	41.5	40.9	39.2	37.4	38.0
Macrolides	27.6	26.9	26.4	26.9	28.2	27.5	26.9	26.8	23.0
Quinolones	18.1	18.3	18.2	18.6	18.3	18.6	19.2	19.2	19.1
Others	3.8	3.5	3.4	3.2	3.2	3.0	3.0	3.2	3.7
Sinusitis (%)									
First-line agent (penicillins)	10.5	10.7	10.8	10.5	11.1	12.1	13.9	15.8	16.5
Non-first-line agent									
Macrolides	37.1	36.3	35.7	35.6	35.6	35.2	35.1	35.0	37.4
Third cephems	33.6	33.6	33.7	33.4	32.6	31.7	30.0	28.1	26.6
Quinolones	15.4	16.2	16.5	17.3	17.7	18.3	18.4	18.8	16.9
Others	3.3	3.3	3.2	3.2	3.0	2.7	2.5	2.4	2.6

FY, fiscal year; Third cephem, third-generation cephalosporins.

### Antibiotic prescriptions among PWH and PWoH

The number of oral antibiotic prescriptions per 1000 population-years, standardized to the 2015 composition of individuals positive for HIV infection, was ∼2.5-fold higher in PWH than in PWoH throughout the study period. Even after excluding the use of trimethoprim-sulfamethoxazole, PWH received approximately twice the number of prescriptions. The penicillin prescription rate was approximately four times higher in PWH than in PWoH (Figure S2). In analyses limited to disease-specific antibiotic prescriptions, PWH had ∼1.5-, 2-, 2.5-, 3- and 12–15 times higher rates of prescriptions than those in PWoH for acute respiratory infections, urinary tract infections, skin infections, acne, pneumonia and sexually transmitted infections, respectively (Table 4).

**Table 4.** Number of antibiotic prescriptions per 1000 population for specific infections in individuals with and without HIV infection

	FY 2012		FY 2014		FY 2016		FY 2018		FY 2020	
	PWoH	PWH	PWoH	PWH	PWoH	PWH	PWoH	PWH	PWoH	PWH
Bronchitis	123.4	170.9	116.6	162.9	116.3	152.0	93.6	125.5	28.8	39.0
Viral respiratory tract infections	110.3	164.4	105.6	156.7	104.8	149.1	85.2	125.3	32.2	52.0
Pharyngitis	106.8	153.1	112.0	166.6	118.2	177.3	106.2	169.0	43.0	71.0
Sinusitis	48.8	58.4	56.3	71.2	62.3	82.6	66.2	90.6	32.0	38.5
Skin infections	23.9	48.9	27.3	63.7	30.1	81.3	32.4	93.6	33.9	87.3
Sexually transmitted infections	2.6	44.5	2.9	39.9	3.0	42.3	3.2	37.9	3.1	37.9
Urinary tract infections	16.3	33.6	17.0	35.1	17.3	36.0	17.2	37.2	16.3	35.1
Pneumonia	6.7	25.6	5.8	18.1	7.5	21.5	6.4	18.2	3.0	8.3
Miscellaneous bacterial infections	9.2	19.1	9.9	19.8	11.7	21.0	11.8	27.3	7.1	17.7
Eye infections	8.6	17.7	8.5	18.0	8.1	16.5	8.0	16.8	6.3	15.6
Acne	4.9	15.6	5.8	17.2	6.5	20.0	7.6	21.2	9.7	23.6
Trauma and burn	10.8	11.2	10.0	8.0	9.2	7.6	8.3	7.7	7.4	7.1
Abdominal infections	2.5	7.7	2.7	9.4	2.9	9.4	2.9	9.4	2.8	8.6
Fever	3.5	6.7	3.6	7.1	4.1	6.6	3.7	7.1	2.3	4.9
Suppurative otitis media	4.0	6.5	4.2	5.4	4.3	5.3	4.3	5.9	2.8	4.5
Influenza	6.0	5.9	6.0	6.5	5.6	6.7	4.0	4.5	0.1	0.4
Gastrointestinal infections	2.5	5.0	2.2	4.2	2.1	5.0	1.9	3.3	1.2	2.3
Others	0.8	1.8	0.8	1.9	0.8	0.9	0.7	1.5	0.4	1.3

FY, fiscal year; HIV, human immunodeficiency virus; PWH, people with HIV infection; PWoH, people without HIV infection.

## Discussion

Japan’s oral antibiotic prescription rate significantly decreased to approximately half in 2020, 5 years after the implementation of the National Action Plan on AMR. However, the quality of antibiotic use showed only a slight improvement, with third-generation cephalosporins, macrolides and fluoroquinolones accounting for ∼70% of oral antibiotic prescriptions. A previous report indicated that the sales volume of oral antibiotics in Japan decreased by 33% in 2021 compared with 2013.^17^ The reduction rate estimated using sales volume was lower than the number of antibiotic prescriptions in this study. Evaluations based on antimicrobial doses, including defined daily dose and sales volume, usually underestimate antibiotic use in children or individuals with renal dysfunction compared with prescription rates.^18^ Therefore, antibiotic use must be evaluated using multiple indicators.

The strength of this study is its inclusion of the longest observation period following the intervention and its comprehensive coverage of patients across all age groups, making it one of the most extensive reports compared with existing studies in Japan.^7–9^ Although various antimicrobial stewardship programmes have effectively reduced antibiotic use,^19^ nationwide evaluations remain limited. A study using a large prescription database in the United Kingdom (UK) reported a 19% reduction in broad-spectrum antibiotic prescriptions in primary care over 2 years following the introduction of a national antimicrobial stewardship programme centred on financial incentives.^20^ Similarly, antimicrobial consumption in South Korea decreased by 30% over 4 years after implementing a national antimicrobial stewardship programme.^21^

Prescription rates were generally higher in western than in eastern Japan, consistent with our previous report.^3^ Similar regional differences in antibiotic prescription rates have also been observed in other countries, including Germany, the United States (US) and the UK.^22–24^ Patient social and economic factors are related to such regional differences.^25^ Interventions promoting appropriate antibiotic use and the implementation of AMR countermeasures should be tailored to regional characteristics.

The proportion of antibiotic prescriptions for respiratory tract infections, another indicator of appropriate use, decreased significantly by ∼15%–20% since the introduction of AMR measures, indicating progress in inappropriate use. Furthermore, during the COVID-19 pandemic in 2020, the downward trend in the proportion of prescriptions did not change significantly. Although diagnostic codes for COVID-19 were not included in this study, some respiratory tract infections diagnosed in 2020 may have represented undiagnosed COVID-19 cases. Given that diagnostic testing for COVID-19 became relatively accessible early in the pandemic and that <500,000 COVID-19 cases were reported in Japan in 2020, the impact on our findings is likely to be minimal. Another study in Japan reported an 11% reduction in the proportion of antibiotic prescriptions for upper respiratory tract infections among adults aged <65 years between 2008 and 2018.^9^

Further studies are needed to evaluate safety outcomes, such as hospitalization associated with reduced outpatient antibiotic prescribing. A US study reported a modest reduction in all-cause hospitalizations following stewardship interventions for respiratory infections.^26^ Conversely, a UK primary care study found no increase in serious invasive infections, but observed small absolute increases in complications, including pneumonia, associated with reduced antibiotic prescribing.^27^

Despite the national action plan, the proportion of first-line antibiotic prescriptions for pharyngitis and sinusitis remained low (<20%), whereas that of broad-spectrum antibiotic prescriptions remained high (∼70%). Studies in other countries have reported greater reductions in broad-spectrum antibiotic use.^24,28^ A comparison of antibiotic prescriptions across 70 countries using 2015 sales data revealed that Japan had the lowest proportion of ‘Access’ antibiotic prescriptions (<40%) among high-income countries.^29^ Future AMR measures in Japan should aim to improve the quality of antibiotic prescriptions by reducing unnecessary use of broad-spectrum antibiotics and increasing the use of narrow-spectrum antibiotics. New financial incentives were introduced in June 2024 to increase the use of ‘Access’ antibiotics in Japan. Furthermore, antibiotic prescription trends after the pandemic must be carefully monitored, considering the temporary impact of the COVID-19 pandemic, and efforts to promote appropriate use should be continued.

In the present study, the antibiotic prescription rate in PWH was ∼2.5-fold higher than that in PWoH; even after excluding sulfamethoxazole-trimethoprim, the rate was consistently more than twice as high. Studies on antibiotic prescription rates in PWH are limited, with no such studies in Japan. A matched-cohort study in the US reported similar rates of visits for acute respiratory tract infections between PWH and PWoH. Although patients with inadequate HIV viral suppression had a 2-fold higher visit rate, compared with PWoH, the overall proportion of antibiotic prescriptions was lower in PWH, likely because PWH managed by HIV clinicians received more guideline-adherent prescriptions with shorter antibiotic durations.^30^ Another study also found that the appropriateness of antibiotic prescriptions was more closely related to the prescriber’s specialty than to HIV status.^31^ The higher rate of antibiotic prescriptions for PWH in the present study is likely due to a higher incidence of specific diseases requiring antibiotics (e.g. sexually transmitted infections and skin infections) and easier access to healthcare due to regular check-ups, rather than a higher prescription rate per visit. As the database used in this study does not include information on physician specialties, we were unable to determine whether PWH were managed by infectious disease specialists. However, in Japan, PWH are typically cared for by infectious disease specialists, which may have influenced the results.

This study has some limitations. First, the study did not include a control group, and the basic interrupted time-series analysis design could not eliminate the potential confounding effects of co-interventions or other concurrent events around the time of the intervention. However, to our knowledge, no other interventions targeting the appropriate use of antimicrobials were implemented in Japan throughout the study period. Second, because we used an administrative claims database, the accuracy of the diagnosis was not validated. Third, antibiotic prescriptions were not entirely linked to diagnoses (e.g. prescribing antibiotics during follow-up visits for the same diagnosis). When linking antibiotic prescriptions to diagnoses, ∼30% of antibiotics were not linked to diagnoses, similar to findings from studies in other countries.^32,33^

In conclusion, the antibiotic prescription rate showed a significant decrease after implementing the National Action Plan in Japan. However, a marked decrease in antibiotic use in 2020 was observed due to the COVID-19 pandemic, suggesting the need to monitor for a rebound in antibiotic usage in the post-COVID period. Because broad-spectrum antibiotics account for 70% of oral antibiotic prescriptions, increased efforts are needed to improve the quality of antibiotic use. Evaluation should continue using multi-faceted quality indicators for antimicrobial stewardship.

## Supplementary Material

dlaf062_Supplementary_Data

## Supplementary data

Figures S1 and S2 and Tables S1 to S3 are available as Supplementary data at *JAC-AMR* Online.
