# Supplementary material for: Significant reduction in antibiotic prescription rates in Japan following implementation of the national action plan on antimicrobial resistance (2016–20): a 9-year interrupted time-series analysis
Source: JAC Antimicrob Resist. 2025 Apr 30;7(3):dlaf062. doi: 10.1093/jacamr/dlaf062 (PMC12041918; doi:10.1093/jacamr/dlaf062)

**Supplementary Table S1. Classification of diagnoses by group and corresponding ICD-10 codes.**

| Diagnosis | ICD-10 codes |
| --- | --- |
| Group 1: Infections for which antibiotics are usually indicated | |
| Abdominal infections | Acute appendicitis (K35), Diverticulitis of intestine (K57), Abscess of anal and rectal regions, intestine, and liver (K61, K630, K750), Peritonitis (K65), Cholecystitis and cholangitis (K800, K801, K803, K804, K810, K819, K830) |
| Miscellaneous bacterial infections | Tuberculosis (A15–A19), Certain zoonotic bacterial diseases (A20–A28), Other bacterial diseases including listeriosis, diphtheria, bartonellosis, erysipelas, and rickettsioses (A30–A37, A39–A49, A75–A79), Bacterial meningitis, encephalitis, and intracranial abscess (G00, G042, G049, G06), Mastoiditis (H70), Infective endocarditis (I33, T826), Acute epiglottitis (J051), Deep neck space infections (J36, J390, J391), Abscess of lung and mediastinum, Pyothorax (J85, J86), Infections of the jaws and mouth (K102, K122), Pyogenic arthritis and osteomyelitis (M00, M462–M465, M86, T845), Pelvic inflammatory diseases (N70–N73, N751, N764, 085), Infections of genitourinary tract in pregnancy (O23, O86), Infection following a procedure, not elsewhere classified (T814), Infections due to cardiac and vascular devices (T827), Infection due to internal prosthetic devices, implants and grafts (T857) |
| Pneumonia | Bacterial pneumonia (J13–J18) |
| Sexually transmitted infections | Infections with a predominantly sexual mode of transmission (A50–A64), Other spirochaetal diseases (A65–A69), Other diseases caused by chlamydiae (A70–A74) |
| Urinary tract infections | Acute pyelonephritis/pyonephrosis (N10, N12, N136), Renal abscess (N151), Kidney infection, unspecified (N159), Acute cystitis (N300), Cystitis, unspecified (N308, N309), Urethritis and urethral abscess (N34), Urinary tract infections, unspecified (N390), Prostatitis and abscess of prostate (N41), Orchitis and epididymitis (N45), Inflammatory disorders of male genital organs, not elsewhere classified (N49), Catheter associated urinary tract infections (T835) |
| Group 2: Infections for which antibiotics are potentially indicated | |
| Acne | Acne (L70) |
| Gastrointestinal infections | Intestinal infectious diseases (A00–A09) |
| Pharyngitis | Streptococcal pharyngitis/tonsillitis (J020, J030), Acute pharyngitis/tonsillitis, unspecified (J029, J039), Scarlet fever (A38) |
| Sinusitis | Acute sinusitis (J01), Chronic sinusitis (J32) |
| Skin, cutaneous and mucosal infections | Infective otitis externa (H600–H603), Infections of other skin and subcutaneous tissue including cellulitis, cutaneous abscess, furuncle, carbuncle, impetigo, acute lymphadenitis, folliculitis, myositis, mastitis, decubitus infection, necrotizing fasciitis (H050, J340, L00–L08, L73, L980, M600, M650–M651, M710–M711, M726, N61, O91), Wound infections (T793) |
| Suppurative otitis media | Suppurative and unspecified otitis media (H66) |
| Group 3: Infections for which antibiotics are rarely indicated | |
| Bronchitis and bronchiolitis | Acute bronchitis (J20), Acute bronchiolitis (J21), Unspecified acute lower respiratory infection (J22), Bronchitis, not specified as acute or chronic (J40) |
| Fever | Fever of unknown origin (R50) |
| Infections of the eye and adnexa | Hordeolum (H00), Blepharitis (H010), Dacryoadenitis(H040), Dacryocystitis (H043–H044), Conjunctivitis (H10), Infections of sclera (H15), Keratitis (H16), Iridocyclitis (H20), Purulent endophthalmitis (H440) |
| Influenza | Influenza (J10, J11) |
| Noninfective diarrhea | Noninfective gastroenteritis and colitis, unspecified (K529) |
| Nonsuppurative otitis media | Nonsuppurative otitis media (H65) |
| Trauma and burn | Open wounds, superficial injuries (S00, S01, S07, S10, S11, S16, S17, S20, S21, S28, S30, S31, S38, S40, S41, S47, S50, S51, S57, S60, S61, S67, S70, S71, S77, S80, S81, S87, S90, S91, S97, T00, T01, T04, T09, T110, T111, T130, T131, T140, T141, T146, and T147), Burns (T200–T203, T210–T213, T220–T223, T230–T233, T240–T243, T250–T253, T290–T293, T300–T303, and T31) |
| Viral pneumonia | Viral pneumonia (J12) |
| Viral upper respiratory infection | Acute nasopharyngitis (common cold) (J00), Acute pharyngitis/tonsillitis due to other specified organisms (J028, J038), Acute laryngitis and tracheitis (J04), Acute obstructive laryngitis (croup) (J050), Acute upper respiratory infections of multiple and unspecified sites (J06), Chronic rhinitis, nasopharyngitis and pharyngitis (J31), Chronic tonsillitis (J350), Chronic laryngitis and laryngotracheitis (J37), Cough (R05) |

**Supplementary Table S2. Annual incidence of antibiotic prescription per 1000 population by prefecture in Japan**.

| Prefecture | Visits with overall antibiotic prescriptions by fiscal year | | | | | | | | |
| --- | --- | --- | --- | --- | --- | --- | --- | --- | --- |
|  | 2012 | 2013 | 2014 | 2015 | 2016 | 2017 | 2018 | 2019 | 2020 |
| Overall | 1221.1 | 1162.8 | 1144.1 | 1164.6 | 1116.0 | 1018.6 | 940.5 | 891.9 | 556.3 |
| Hokkaido | 968 | 945 | 894 | 921 | 880 | 810 | 746 | 702 | 451 |
| Aomori | 1230 | 1170 | 1108 | 1140 | 1115 | 989 | 895 | 828 | 537 |
| Iwate | 1013 | 969 | 932 | 956 | 909 | 831 | 759 | 711 | 488 |
| Miyagi | 1219 | 1103 | 1053 | 1083 | 1025 | 931 | 856 | 796 | 498 |
| Akita | 1134 | 1083 | 1035 | 1049 | 1022 | 918 | 840 | 760 | 516 |
| Yamagata | 1010 | 954 | 928 | 932 | 894 | 829 | 766 | 725 | 488 |
| Fukushima | 1241 | 1124 | 1105 | 1120 | 1063 | 950 | 863 | 798 | 500 |
| Ibaraki | 1089 | 1035 | 1001 | 1026 | 989 | 901 | 829 | 787 | 489 |
| Tochigi | 1160 | 1107 | 1074 | 1103 | 1054 | 941 | 858 | 805 | 517 |
| Gunma | 1222 | 1148 | 1126 | 1127 | 1080 | 960 | 879 | 832 | 504 |
| Saitama | 1068 | 1009 | 991 | 1015 | 967 | 887 | 824 | 772 | 468 |
| Chiba | 1063 | 1012 | 991 | 1018 | 972 | 878 | 803 | 748 | 455 |
| Tokyo | 1319 | 1261 | 1232 | 1256 | 1183 | 1093 | 1020 | 961 | 570 |
| Kanagawa | 1138 | 1081 | 1062 | 1081 | 1023 | 944 | 885 | 832 | 508 |
| Niigata | 1183 | 1142 | 1108 | 1089 | 1050 | 920 | 874 | 802 | 512 |
| Toyama | 1152 | 1104 | 1060 | 1078 | 1029 | 910 | 854 | 829 | 487 |
| Ishikawa | 1137 | 1094 | 1065 | 1086 | 1045 | 934 | 911 | 851 | 559 |
| Fukui | 1192 | 1159 | 1100 | 1123 | 1117 | 979 | 969 | 919 | 585 |
| Yamanashi | 1159 | 1079 | 1084 | 1104 | 1073 | 997 | 893 | 877 | 537 |
| Nagano | 1046 | 972 | 995 | 1009 | 967 | 874 | 816 | 783 | 474 |
| Gifu | 1485 | 1377 | 1342 | 1364 | 1301 | 1161 | 1054 | 986 | 575 |
| Shizuoka | 1219 | 1127 | 1128 | 1139 | 1075 | 947 | 858 | 822 | 525 |
| Aichi | 1293 | 1210 | 1207 | 1228 | 1172 | 1055 | 972 | 907 | 557 |
| Mie | 1289 | 1237 | 1229 | 1251 | 1185 | 1088 | 998 | 947 | 582 |
| Shiga | 1100 | 1039 | 1038 | 1046 | 994 | 919 | 848 | 799 | 503 |
| Kyoto | 1109 | 1066 | 1063 | 1088 | 1032 | 942 | 864 | 833 | 510 |
| Osaka | 1306 | 1265 | 1267 | 1316 | 1249 | 1172 | 1084 | 1037 | 633 |
| Hyogo | 1225 | 1183 | 1164 | 1196 | 1129 | 1046 | 968 | 924 | 574 |
| Nara | 1236 | 1170 | 1170 | 1202 | 1135 | 1044 | 956 | 905 | 546 |
| Wakayama | 1263 | 1209 | 1206 | 1216 | 1185 | 1130 | 1020 | 995 | 612 |
| Tottori | 1267 | 1190 | 1135 | 1173 | 1103 | 985 | 885 | 853 | 555 |
| Shimane | 1270 | 1219 | 1221 | 1210 | 1165 | 1008 | 932 | 882 | 562 |
| Okayama | 1335 | 1288 | 1247 | 1277 | 1210 | 1122 | 1040 | 987 | 621 |
| Hiroshima | 1434 | 1346 | 1347 | 1349 | 1292 | 1188 | 1076 | 1028 | 666 |
| Yamaguchi | 1292 | 1202 | 1207 | 1207 | 1221 | 1093 | 999 | 949 | 630 |
| Tokushima | 1482 | 1319 | 1334 | 1356 | 1345 | 1252 | 1134 | 1098 | 679 |
| Kagawa | 1401 | 1306 | 1319 | 1362 | 1286 | 1183 | 1056 | 1007 | 642 |
| Ehime | 1399 | 1322 | 1299 | 1347 | 1312 | 1189 | 1093 | 1060 | 658 |
| Kochi | 1276 | 1182 | 1161 | 1204 | 1211 | 1092 | 987 | 973 | 633 |
| Fukuoka | 1336 | 1295 | 1285 | 1272 | 1260 | 1148 | 1064 | 1026 | 653 |
| Saga | 1345 | 1297 | 1287 | 1291 | 1307 | 1168 | 1076 | 1039 | 717 |
| Nagasaki | 1312 | 1259 | 1264 | 1262 | 1239 | 1133 | 1014 | 988 | 658 |
| Kumamoto | 1413 | 1393 | 1366 | 1353 | 1360 | 1210 | 1090 | 1043 | 700 |
| Oita | 1469 | 1390 | 1358 | 1385 | 1390 | 1248 | 1158 | 1131 | 742 |
| Miyazaki | 1402 | 1376 | 1333 | 1399 | 1398 | 1271 | 1188 | 1159 | 795 |
| Kagoshima | 1288 | 1247 | 1219 | 1229 | 1203 | 1091 | 1026 | 995 | 699 |
| Okinawa | 1022 | 1045 | 1014 | 994 | 990 | 929 | 872 | 868 | 583 |

**Supplementary Table S3. Proportions of oral antibiotic prescriptions during outpatient visits for specific infections.**

|  | FY 2012 | FY 2013 | FY 2014 | FY 2015 | FY 2016 | FY 2017 | FY 2018 | FY 2019 | FY 2020 |
| --- | --- | --- | --- | --- | --- | --- | --- | --- | --- |
| Group 2 |  |  |  |  |  |  |  |  |  |
| Pharyngitis | 56.9% | 55.8% | 55.0% | 54.3% | 52.1% | 47.8% | 43.9% | 42.1% | 37.7% |
| Sinusitis | 55.2% | 56.1% | 55.7% | 56.0% | 55.5% | 53.2% | 51.3% | 51.1% | 41.9% |
| Gastrointestinal infections | 36.3% | 37.6% | 39.2% | 37.7% | 36.5% | 36.2% | 34.7% | 33.9% | 41.2% |
| Skin infections | 51.4% | 50.6% | 49.9% | 49.2% | 48.6% | 47.4% | 47.1% | 46.7% | 46.5% |
| Suppurative otitis media | 68.7% | 69.3% | 70.1% | 70.4% | 70.6% | 69.9% | 69.5% | 68.9% | 61.7% |
| Acnes | 37.4% | 37.0% | 36.3% | 33.7% | 32.4% | 31.7% | 31.8% | 31.4% | 31.2% |
| Group 3 |  |  |  |  |  |  |  |  |  |
| Bronchitis | 44.7% | 42.2% | 40.1% | 38.7% | 36.6% | 32.2% | 28.4% | 26.8% | 22.6% |
| Viral upper respiratory infections | 28.5% | 26.9% | 25.7% | 24.9% | 23.4% | 20.3% | 17.6% | 16.5% | 13.9% |

FY, fiscal year.

Groups 2 and 3: infections for which antibiotics are potentially and rarely indicated, respectively.

Supplementary Figure S1. Trends and interrupted time-series analyses of the proportions of antibiotic prescriptions for specific infections in Japan between fiscal years 2012 and 2019, excluding the coronavirus disease (COVID-19) pandemic period. Antibiotic prescriptions for (a) pharyngitis, (b) sinusitis, (c) bronchitis, and (d) viral upper respiratory infections. Vertical line: timing of National Action Plan implementation in April 2016. Gray background color: post-intervention period. Dots: monthly numbers of antibiotic prescriptions per 100 visits for each infection (proportion of prescribing). Solid lines: estimated model. Dashed lines: counterfactual scenario without the intervention. The incidence rate ratio (IRR) and its 95% confidence interval (CI) of the pre-intervention trend were estimated using Poisson regression to reflect changes in the incidence rate between 2012 and 2016. The impact of intervention represents the additional annual reduction in the incidence rate ratio during the post-intervention period (2016–2019).

Supplementary Figure S2. Trends in the number of oral antibiotic prescriptions per 1,000 population-years in people with human immunodeficiency virus (HIV) (PWH) and without HIV (PWoH) infection. The rates among PWH were standardized to the 2015 PWH population for comparison with PWoH, who were also standardized to the 2015 PWH population.


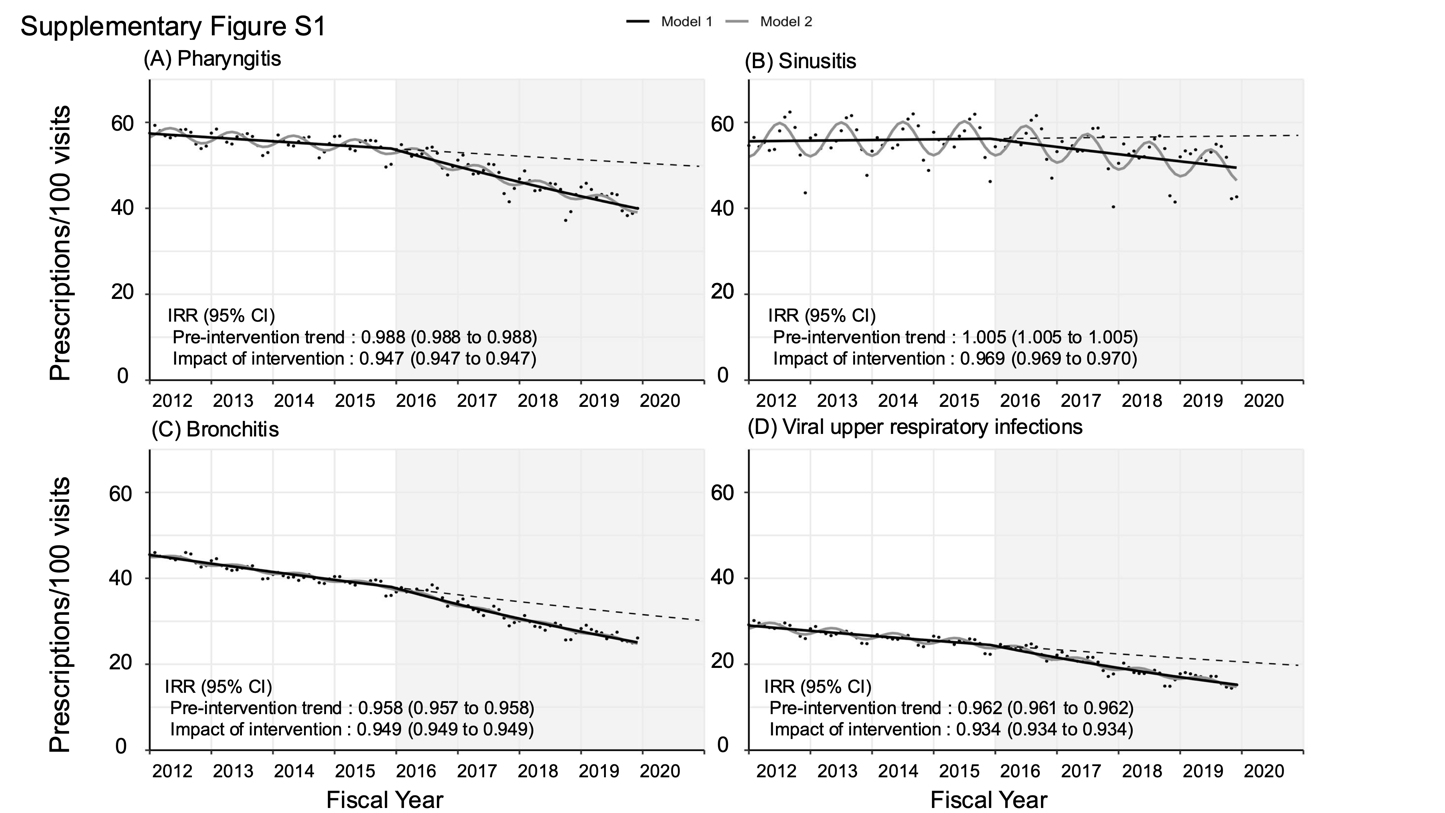


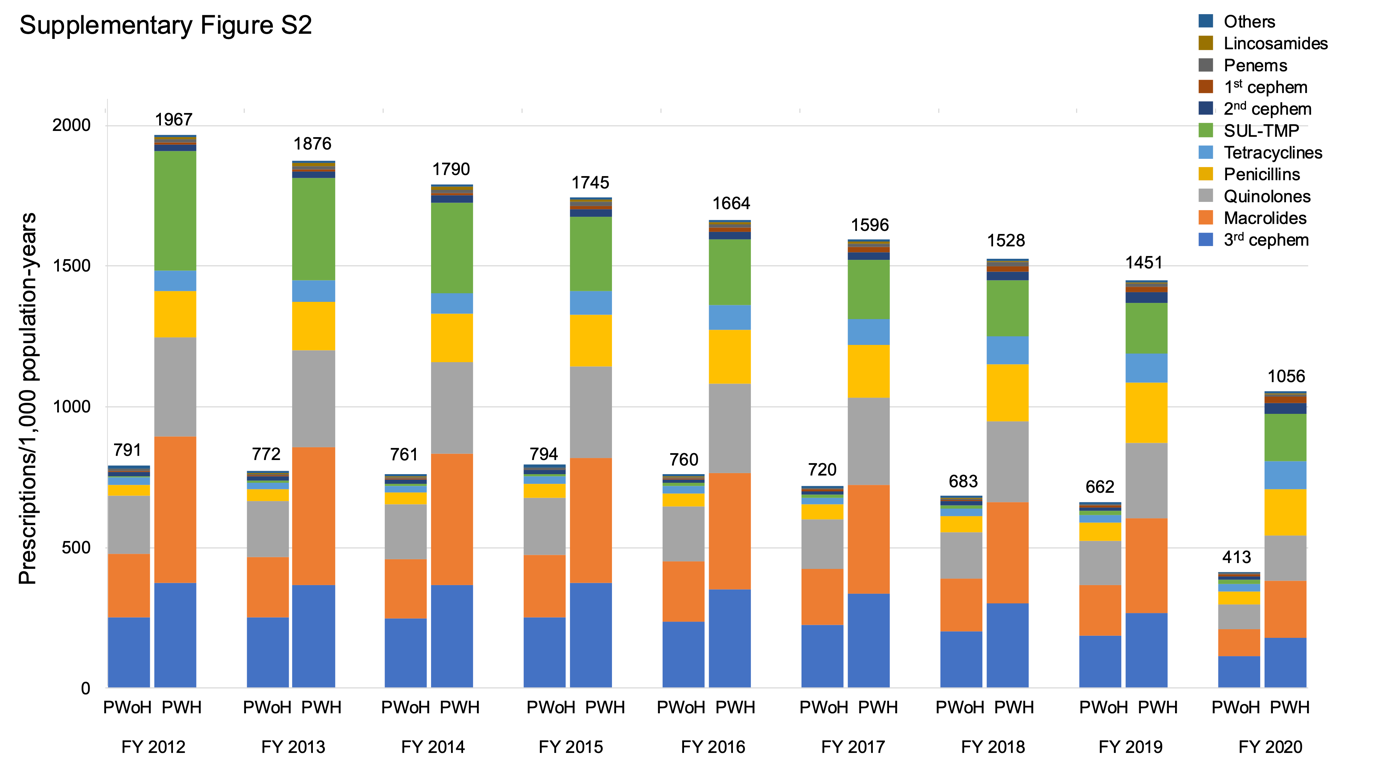

Supplement: dlaf062_Supplementary_Data [file dlaf062_supplementary_data.docx]
